# Synthesis, Characterisation, and Evaluation of a Cross-Linked Disulphide Amide-Anhydride-Containing Polymer Based on Cysteine for Colonic Drug Delivery

**DOI:** 10.3390/ijms141224670

**Published:** 2013-12-18

**Authors:** Vuanghao Lim, Kok Khiang Peh, Shariza Sahudin

**Affiliations:** 1 Integrative Medicine Cluster, Advanced Medical and Dental Institute, Universiti Sains Malaysia, Bertam, 13200 Kepala Batas, Penang, Malaysia; 2 Discipline of Pharmaceutical Technology, School of Pharmaceutical Sciences, Universiti Sains Malaysia, 11800 Minden, Penang, Malaysia; E-Mail: kkpeh@usm.my; 3 Faculty of Pharmacy, UiTM Puncak Alam Campus, Bandar Puncak Alam, 42300 Selangor, Malaysia

**Keywords:** disulphide cross-linked polymer, synthesis, colon drug delivery, amide-anhydride, cysteine

## Abstract

The use of disulphide polymers, a low redox potential responsive delivery, is one strategy for targeting drugs to the colon so that they are specifically released there. The objective of this study was to synthesise a new cross-linked disulphide-containing polymer based on the amino acid cysteine as a colon drug delivery system and to evaluate the efficiency of the polymers for colon targeted drug delivery under the condition of a low redox potential. The disulphide cross-linked polymers were synthesised via air oxidation of 1,2-ethanedithiol and 3-mercapto-*N*-2-(3-mercaptopropionamide)-3-mercapto propionic anhydride (trithiol monomers) using different ratio combinations. Four types of polymers were synthesised: P10, P11, P151, and P15. All compounds synthesised were characterised by NMR, IR, LC-MS, CHNS analysis, Raman spectrometry, SEM-EDX, and elemental mapping. The synthesised polymers were evaluated in chemical reduction studies that were performed in zinc/acetic acid solution. The suitability of each polymer for use in colon-targeted drug delivery was investigated *in vitro* using simulated conditions. Chemical reduction studies showed that all polymers were reduced after 0.5–1.0 h, but different polymers had different thiol concentrations. The bacterial degradation studies showed that the polymers were biodegraded in the anaerobic colonic bacterial medium. Degradation was most pronounced for polymer P15. This result complements the general consensus that biodegradability depends on the swellability of polymers in an aqueous environment. Overall, these results suggest that the cross-linked disulphide-containing polymers described herein could be used as coatings for drugs delivered to the colon.

## 1. Introduction

The efficiency of drug delivery of a pharmaceutical dosage can be maximised by employing the strategy of site-specific drug delivery [1]. Treatment of medical conditions such as inflammatory bowel diseases (IBD and Crohn’s disease) or colon cancer would benefit from improved delivery of drugs selectively to the colon [2]. Oral absorption of peptide and protein drugs (e.g., biotechnological preparations) is known to be limited due to the harsh environments of the stomach and intestine. Hence, the colon, with its low peptidase activity and longer transit times, might provide the best site for the absorption of peptide and protein drugs [3]. Delivery to the colon would provide an alternative and better means for the administration of peptide and protein drugs into the human system.

Polymers, which degrade either in the presence of bacterial enzymes [4] or under the low oxidation potential in the colon [5], are interesting prospects for drug delivery due to their selectivity. Examples include the azo polymer and polysaccharide polymers [1,3]. The degradation of disulphide compounds in response to the low redox potential of the colon has gained much attention [5,6]. Linear redox-sensitive polymers containing azo and/or disulphide linkages in the backbone have been synthesised [6]. Although studies of linear chain disulphide polymers have been performed [6], no studies of cross-linked disulphide polymers as dosage form coating materials (capsules or tablets) have been published. The author also specifically dismissed any role of cross-linked disulphide polymers in achieving site specific colonic delivery due to their insolubility. Studies on the synthesis of disulphide cross-linked poly(amidoamine) dendrimers [7] and nanocapsules [8] have been reported as carrier in effective drug delivery. In general, cross-linked disulphide polymers are more insoluble than linear chain disulphide polymers [9–11]. Wilkes (1981) [12] stated that chemical cross-linking whereby polymer chains are bonded together by either ionic or covalent bonds to form a polymer network would limit movement of the polymer chain, hence restricting the exposure of amines and carboxyl group. The comparison of hydrodynamic volumes of linear and branched chain polymers with similar molecular weight was made, and it was found that the latter possessed lower hydrodynamic volumes due to its more compact structure which in turn limits the exposure to water or solvent. Nevertheless, if the desired polymer insolubility can be achieved, then this insolubility would be a great advantage in protecting the drug or formulation during its transit through the harsh conditions of the stomach and small intestine.

In principle, when polymers reach the colon, the disulphide bridges [13] will be broken due to the reducing environment of the colon [4,5], thereby releasing the drug. In this study, branch chained disulphide polymers based on the amino acid cysteine were synthesised. The reason for using cysteine [14] is that it is not foreign to the body, as it is an essential amino acid synthesised by the body. Therefore, side effects that might occur after the degradation of the cysteine-based polymer should be reduced. The polymers were characterised using various spectroscopic methods and were validated using chemical reduction studies. The susceptibility of the polymers in a low redox environment was tested in simulated conditions of the colon using *Bacteroides fragilis*. Incubations were run at three different intervals of 5, 30 and 180 h. The results obtained present an important contribution to the possibility of using new polymers based on disulphide backbone as dosage form coating materials for pharmaceutical products such as capsules or tablets in a colon targeting drug delivery system.

## 2. Results and Discussion

### 2.1. Synthesis

**Scheme 1.**
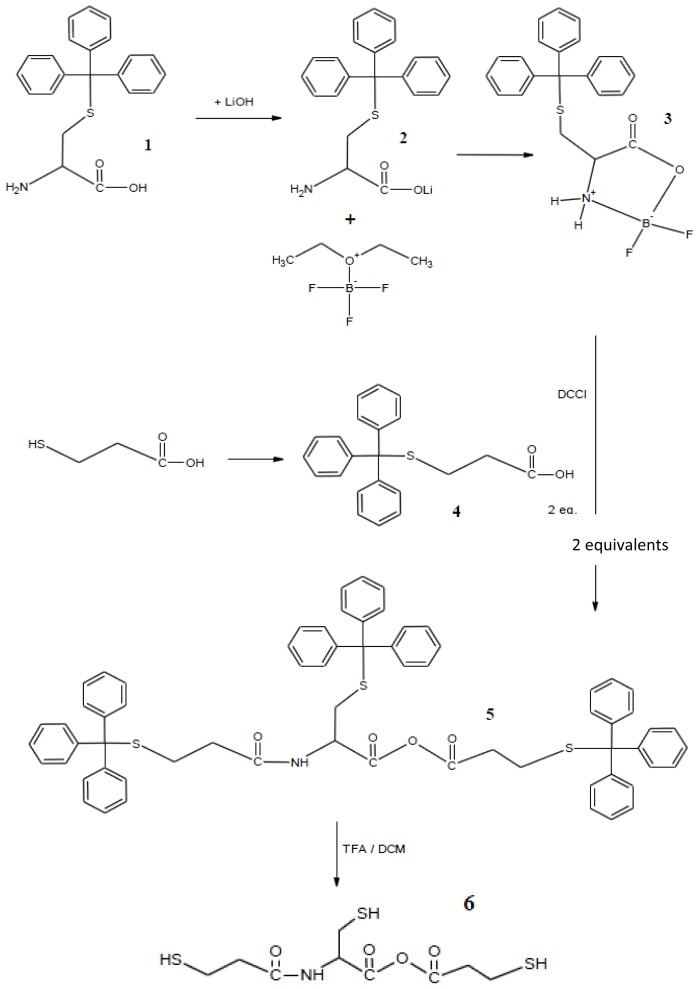
Synthetic routes for the preparation of trithiol cysteine derivative monomer **6**.

The synthesis of trithiol cysteine derivative monomer **6** is presented in Scheme 1. Compound **3** was synthesised in low yield from **1** and **2**; this compound remained stable for at least 5 d under stringent dry conditions (opening of the oxazolidinone ring). This oxazolidinone compound was reacted with two equivalents of **4** in a coupling synthesis to produce compound **5**. Compound **6** was prepared via deprotection of the trityl protection groups.

### 2.2. Physical Characterisation of Disulphide Cross-Linked Polymers

Detection of sodium nitroprusside showed a negative test for thiols at the termination of each oxidative reaction, indicating successful disulphide bond formation. The formation of disulphide bonds was also supported by the emergence of the *S*–*S* peak in Raman spectrometry, as described in Section 2.3. The solubility of the synthesised polymers was determined in water, dichloromethane (DCM), and dimethyl sulphoxide (DMSO) (Table 1).

Oxidative polymerisation [14] using DMSO yielded polymers with “tight” or “loose” polymeric network from various molar combinations of the trithiol or dithiol monomers. A “tight” polymer structure would be formed from the oxidation of trithiol monomer, whereas a “loose” polymer structure would be formed from the oxidation of a combination of tri/di monomers [9,15] The Fourier-transform infrared (FTIR) spectra for the four synthesised disulphide cross-linked polymers with different ratios are shown below:

Polymer P10: FTIR (KBr disc) = 3432 cm^−1^ (–NH stretch), 2925 cm^−1^ (–CH_2_–), 1704 cm^−1^ (–CO–O–CO–), 1654, 1593 cm^−1^ (–NHCO–), 1180 cm^−1^ (C–O stretch).
Polymer P11: FTIR (KBr disc) = 3408 cm^−1^ (–NH stretch), 2938 cm^−1^ (–CH_2_–), 1708 cm^−1^ (–CO–O–CO–), 1654, 1593 cm^−1^ (–NHCO–), 1189 cm^−1^ (C–O stretch).
Polymer 151: FTIR (KBr disc) = 3405 cm^−1^ (–NH stretch), 2917 cm^−1^ (–CH_2_–), 1687 cm^−1^ (–CO–O–CO–), 1654 cm^−1^ (–NHCO–), 1176 cm^−1^ (C–O stretch).
Polymer 15: FTIR (KBr disc) = 3307 cm^−1^ (–NH stretch), 2913 cm^−1^ (–CH_2_–), 1704 cm^−1^ (–CO–O–CO–), 1597 cm^−1^ (–NHCO–), 1185 cm^−1^ (C–O stretch).

The oxidative polymerisation of trithiol monomers alone (polymer P10) yielded a polymer insoluble in water, DCM, and DMSO. Similar results were observed for polymers P11 and P151. However, a polymer soluble in DMSO was obtained with the feed molar ratio of trithiol:dithiol = 1:5 (polymer P15). This result suggests that polymer solubility can be increased by increasing the molar ratio of dithiol. Sim *et al.* [15,16] also performed oxidative polymerisation using iron (III) chloride as an oxidant. They found that performing the oxidation–polymerisation of the 4-*N*-butyltriphenylamine monomer within a short or long period of time resulted in a low or high yield of the insoluble portion, respectively. The insolubility of the polymer produced via the oxidative polymerisation of *N*-butyl-*N*,*N*-diphenylamine and *N*-butylphenyl-*N*,*N*-diphenylamine can be increased by increasing the temperature and amount of oxidant (*i.e*., FeCl_3_) [16].

DMSO oxidation was used because of its vital function as a solvent for the trithiol monomer, which makes the polymerisation process more effective. DMSO is a mild oxidising agent [17] for simple organic thiols; it facilitates the production of H_2_O and dimethyl sulphide as harmless by-products [17–19]. Among the four synthesised polymers, polymer P10 had the most tightly cross-linked polymeric network because of the oxidation of the trithiol monomer alone. Polymers P11, P151, and P15 were looser than polymer P10 because they were synthesised from different molar combinations of trithiol and dithiol monomers. The tight polymer (P10) appeared as a rugged white solid, whereas the loose polymer (P15) appeared as a yellowish white powder. The other two loose polymers (P11 and P151) were white solids. Thus, polymerisation might have taken place via the precipitation of an insoluble product of the reaction mixture upon exposure to DMSO in open air. The detection of sodium nitroprusside showed a negative test for thiols at the termination of each oxidative reaction. The formation of disulphide bond was also supported by the emergence of the *S*–*S* peak in Raman spectroscopy.

### 2.3. Raman Spectroscopy

**Figure 1.**
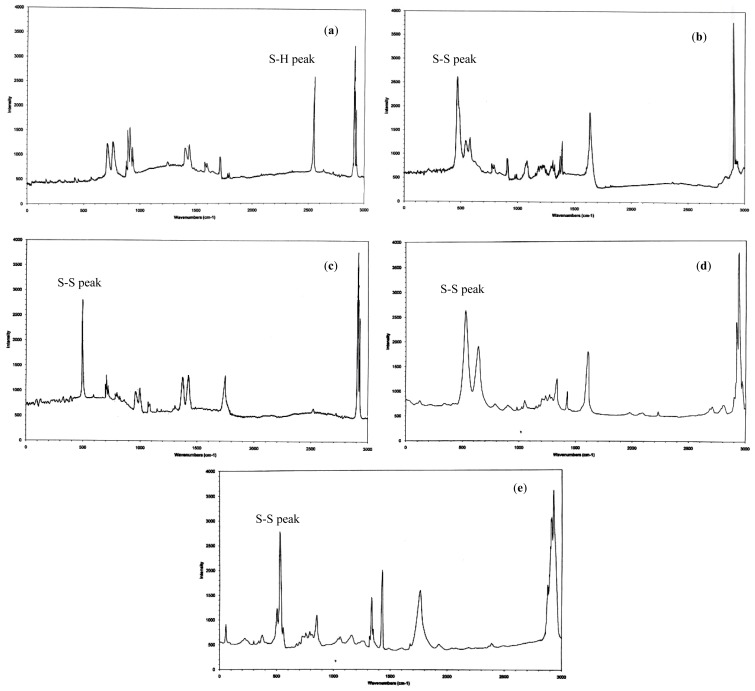
Raman spectra of (**a**) the trithiol monomer and polymers (**b**) P10; (**c**) P11; (**d**) P151; and (**e**) P15.

Raman spectroscopy is an important method for determining the presence of disulphide bonds. The results demonstrated the successful oxidation of the trithiol monomers using DMSO as the oxidising agent. The Raman spectra obtained for the monomer showed a strong *S*–*H* peak at 2553 cm^−1^; however, this peak was not observed in the spectra of the polymers (Figure 1). The disulphide peak *S*–*S* was clearly observed in the spectra of all four disulphide cross-linked polymers; this peak was located in the region of 430–550 cm^−1^. Raman spectroscopy results also showed the presence of carbon sulphide bonding [υ(CS)], as indicated by the peaks in the region of 630–790 cm^−1^.

### 2.4. Morphological Aspects and Energy Dispersive X-ray (EDX) Micrographs

**Figure 2.**
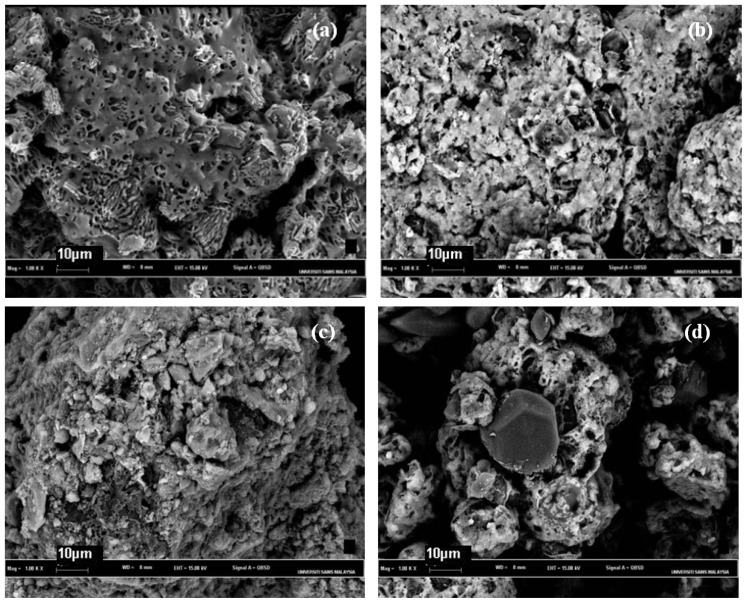
Scanning electron micrographs at 1000× of polymers (**a**) P10; (**b**) P11; (**c**) P151, and (**d**) P15.

Figure 2 shows scanning electron micrographs (SEM) of the four polymers. All polymers in this experiment exhibited different surface morphologies. Basically, the synthesised polymers using different ratios were rough. This finding suggests that the polymers were unevenly deposited compared with the monomer. Polymer P10 presented a rougher surface compared with the other polymers. This type of surface morphology might be assumed to be related to the tight polymeric network, where the oxidation of trithiol monomer alone contributed to the more compact zone within the polymer itself.

Polymer P15 exhibited an uneven porous surface (Figure 2d), likely due to its loose disulphide polymer network. At 1000× magnification, polymers P10, P11, and P151 were more compact than polymer P15. This observation is in agreement with Diaz *et al.* [20], who studied poly[bis(2-aminophenyl)disulfide] (DSDA) and poly[bis(2-aminophenyl)diselenide] (DSeDA) polymers. They found that DSeDA, with its tight polymeric network, was more rugged compared with DSDA. This observation further indicates that longer oxidative polymerisation results in more compact features visible under SEM compared to shorter oxidative polymerisation.

**Figure 3.**
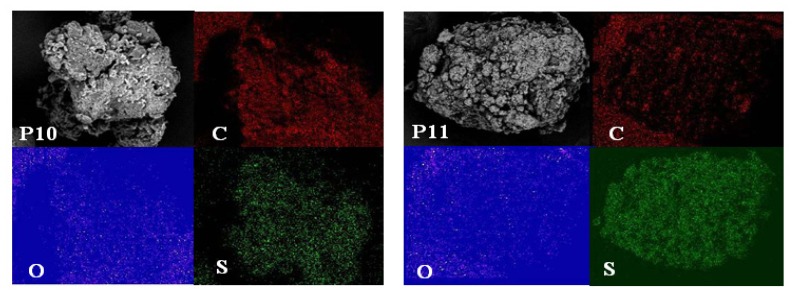

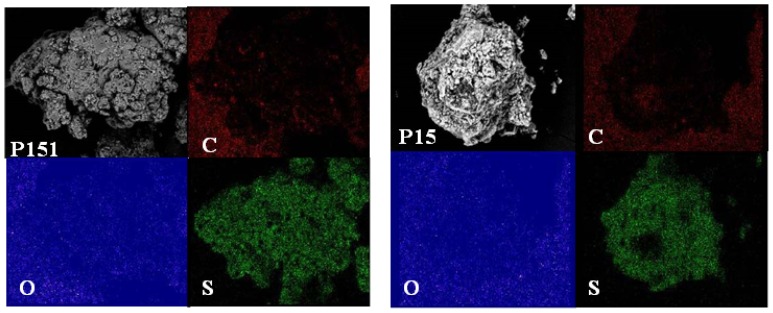
SEM micrographs of polymers P10, P11, P151, and P15 and elemental maps for carbon (C), oxygen (O), and sulphur (S) for the same regions obtained by EDX mapping.

EDX spectroscopy of the polymers, which provides *in situ* chemical analysis of the bulk, clearly showed carbon, oxygen, and sulphur as constituents of the synthesised polymers. These results were also supported by the elemental mapping of the polymers (Figure 3). Elemental mapping techniques are excellent tools because they do not require the insertion of a staining agent [21]. The oxygen map of all four polymers exhibited a similar intensity distribution on the sulphur map. This result indicates that all four synthesised polymers reacted homogenously. The intensity distribution of sulphur in the more loose polymers (P11, P151, and P15) was higher than that in the tight polymer (P10).

**Figure 4.**
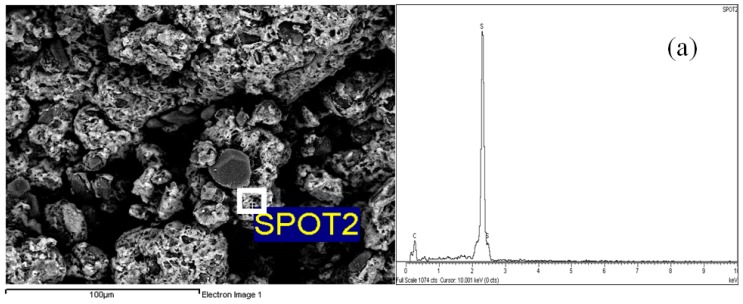

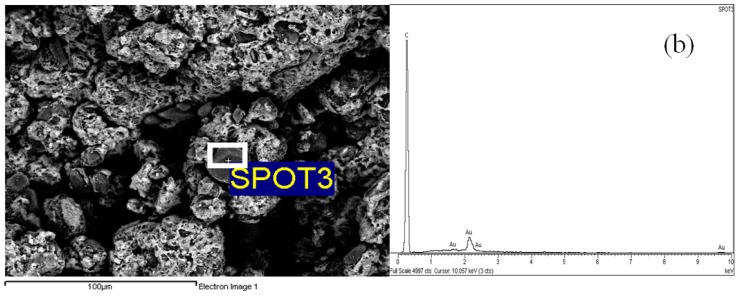
SEM-EDX maps showing the actual positions of the distributions of (**a**) sulphur and (**b**) carbon in polymer P15.

The SEM micrographs of polymer P15 (500× magnification) were selected to investigate the actual positions of the elemental distributions. The brighter layers of the surface in the micrograph can be attributed to sulphur (spot 2), whereas the darker (grey) spots can be attributed to carbon (spot 3) (Figure 4).

### 2.5. Chemical Reduction Studies

#### 2.5.1. Chemical Reduction of Cystamine

A calibration plot of absorbance against concentration of thiols was conducted using cystamine as the standard. The curve was linear and fitted to pass through the origin. The correlation coefficients obtained for the calibration curve was high, with an *R*^2^ value of 0.9994 (graph not shown). Table 2 shows that the cystamine was reduced by zinc/acetic acid after 20 min of reaction. The excess zinc in the reaction was successfully removed by filtration through a Pasteur pipette containing pre-inserted cotton wool.

#### 2.5.2. Chemical Reduction of Disulphide Cross-Linked Polymers

**Figure 5.**
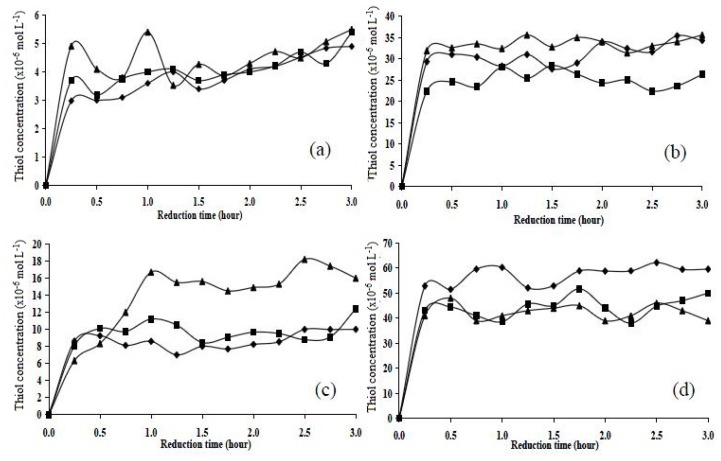
Chemical reduction of polymers (**a**) P10; (**b**) P11; (**c**) P151; and (**d**) P15 using zinc/acetic acid in three batches (◆ batch 1, ■ batch 2, ▲ batch 3).

The assay for thiols was carried out for 3 h and the results were tabulated (Figure 5). The disulphide content, from the molar ratio of monomers used in the synthesis, was found to be highest in polymer P15 (approx. 50 × 10^−6^ mol L^−1^), followed by polymers P11 (approx. 28 × 10^−6^ mol L^−1^), P151 (approx. 28 × 10^−6^ mol L^−1^), and P10 (approx. 4 × 10^−6^ mol L^−1^). The maximum reduction occurred after 1 h, and the plateau was reached at 3 h. Chemical reduction studies showed that all polymers were reduced after 0.5–1.0 h, but they contained different thiol concentrations.

#### 2.6. Incubation of Polymers with *B. fragilis* for 5, 30, and 180 h

Pure cultures of *B. fragilis* cell pellets in SØrensen’s phosphate buffer at pH 7.4 were used for the bacterial degradation studies. Bragger *et al.* (1997) [4] used *B. fragilis* for the reduction of azo cross-linked polymers for colon-specific drug delivery. In this study, the polymers investigated were reduced in such a bacterial culture. In the current study, the *B. fragilis* culture was used to generate the low redox environment of the colon. The incubation was performed in SØrensen’s phosphate buffer rather than cooked meat or Wilkens-Chalgren Anaerobic Broth (WCAB) because it was necessary to have a clear sample solution to detect thiol content spectrophotometrically using Ellman’s reagent.

**Figure 6.**
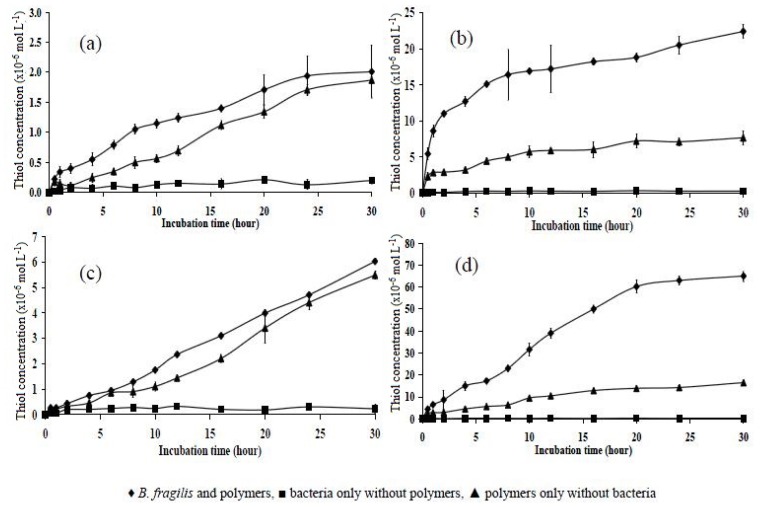
Thiol concentration as a function of incubation time in phosphate buffer medium over a 5 h time period in polymers (**a**) P10; (**b**) P11; (**c**) P151; and (**d**) P15. Mean ± SD, *n* = 6.

The initial incubations were carried out with the four polymers (P10, P11, P151, and P15) for a period of 5 h (Figure 6). The first three test polymers showed a slow response to the Ellman’s test for thiol (development of an intense yellow colour). Thiol was detected at a very low concentration after 1 h of incubation. The concentrations of thiol in polymers P10, P11, and P151 were 0.49 × 10^−6^, 0.15 × 10^−6^, and 0.32 × 10^−6^ mol L^−1^, respectively. For each of the three polymers, there was little difference between the amount of thiol detected when incubated with bacteria/phosphate buffer or buffer alone. Therefore, these results indicate that polymers P10, P151, and P11 were too tight to be reduced by the bacteria. However, after 3.5 h of incubation, the data for polymer P11 began to differ for the different conditions; a thiol concentration of approximately 12.2 × 10^−6^ mol L^−1^ was detected in the bacterially incubated polymer suspension, whereas the value was 3.34 × 10^−6^ mol L^−1^ for the polymer incubated in phosphate buffer alone (Figure 6b).

The same trend was found for polymer P15, as the difference in the thiol concentration between the bacteria/phosphate buffer incubated polymer and the polymer incubated in phosphate buffer only was relatively high (Figure 6d). The thiol concentration detected in polymer P15 incubated in the bacteria/phosphate buffer showed a steady increase, with a slight drop after 4.5 h of incubation, but it increased again at 5 h. However, because the substrates were insoluble polymers and incubation times between 12 and 24 h have been reported to effect reduction of azo polymers incubated in bacterial culture [22], 5 h was not a long enough incubation time to allow conclusions or judgments to be made about the reduction of disulphide cross-linked polymers by *B. fragilis*.

**Figure 7.**
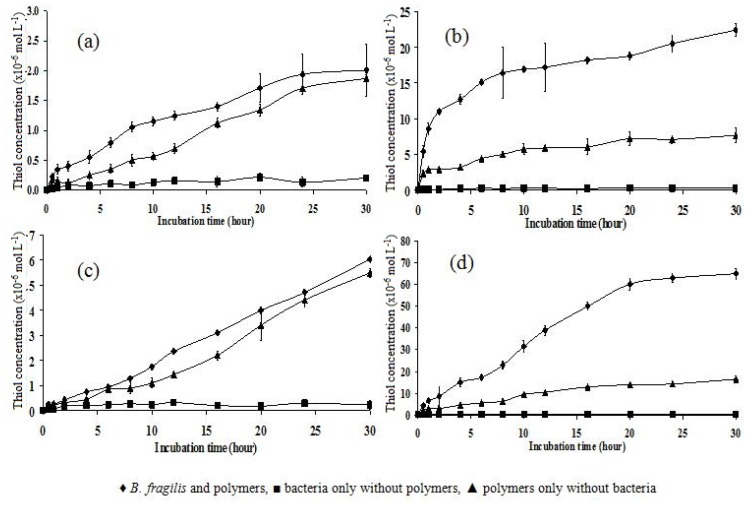
Thiol concentration as a function of incubation time in phosphate buffer medium over a 30 h time period in polymers (**a**) P10; (**b**) P11; (**c**) P151; and (**d**) P15. Mean ± SD, *n* = 6.

Therefore, the experiment was repeated using a longer (*i.e*., 30 h) incubation time (Figure 7). The data obtained for polymers P10 and P151 showed little difference between the amount of thiol detected from polymers incubated with bacteria/phosphate buffer or buffer alone. Both polymers in both media exhibited an increase in thiol concentration over time. However, the thiol concentrations detected in polymers P10 and P151 were low compared to those detected in polymers P11 and P15, for which the highest values were 20.1 × 10^−6^ and 60.3 × 10^−6^ mol L^−1^, respectively, after 30 h of incubation. This indicates that the tight polymers (P10 and P151) were insoluble in water and reduction by bacteria was unlikely to occur in such dense polymers. The curves for the polymer P11 data were similar for the polymers incubated in bacteria/phosphate buffer and the polymers incubated in phosphate buffer alone, but the thiol concentration of the latter was lower.

The reduction by bacteria was clearly visible in the data for polymer P15, as the thiol concentration increased over the 30 h time period. Theoretically, polymer P15 has the loosest polymer network, which might be more susceptible to bacterial degradation. This premise is supported by the fact that the thiol concentration of polymer P15 incubated in bacteria/phosphate buffer had the highest value among all polymers tested (60.43 × 10^−6^ mol L^−1^).

**Figure 8.**
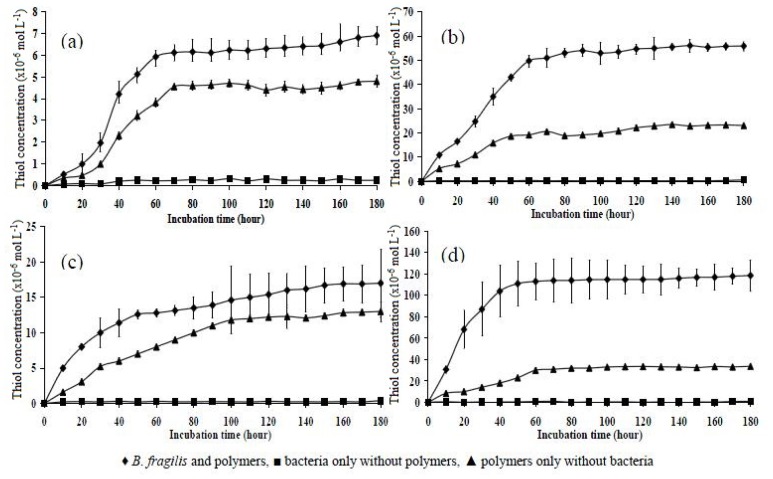
Thiol concentration as a function of incubation time in phosphate buffer medium over a 180 h time period in polymers (**a**) P10; (**b**) P11; (**c**) P151; and (**d**) P15. Mean ± SD, *n* = 6.

In a third experiment, the four polymers were incubated for an extended incubation time of 180 h (Figure 8) to observe the trends of thiol concentration over a longer period of time. There was no difference in the data obtained for the control (polymer in phosphate buffer) and the tests (polymers in bacterial culture) for polymers P10 and P151. The thiol concentrations plotted as a function of time showed that the thiol level in polymer P10 increased until 70 h of incubation and then remained virtually the same for the remainder of the experiment. The dense polymer P151 showed no significant reduction of disulphide from the thiol concentrations obtained, as the data for the control polymers in phosphate buffer and the bacterially incubated polymers were almost superimposed. There was virtually no thiol detected in the bacterial suspension alone in all four treatments. For polymers P11 and P15, a steep rise in the concentration of thiol detected in the bacterially incubated polymers occurred over time, with values increasing from approximately 11.9 × 10^−6^ to 49.7 × 10^−6^ mol L^−1^ in polymer P11 and 30.7 × 10^−6^ to 116.3 × 10^−6^ mol L^−1^ in polymer P15.

After 40 h, a plateau in thiol concentration (approximately 110 × 10^−6^ mol L^−1^) was reached. There was clearly more thiol detected from the bacterially incubated polymers than in phosphate buffer alone, which corroborates the premise that polymer P15 was more susceptible to the degradation of *B. fragilis* in SØrensen’s phosphate buffer. The results of statistical comparisons of thiol concentration of various molar combinations of polymers at different incubation times are presented in Tables 3 and 4.

## 3. Experimental Section

In the following experiments, a calcium chloride drying tube was used as a protection against moisture during the reaction process. Nitrogen gas was employed in all reactions requiring an inert atmosphere. All glassware used as a reaction vessel was oven dried for 48 h. The reactions began by washing with 2 mol L^−1^ citric acid and saturated sodium bicarbonate solution to remove any unreacted compounds (e.g., free acid, base) in the reaction mixture. Anhydrous solutions were obtained by submerging molecular sieve granules for at least 24 h. All samples were stored in a desiccator and were dried over anhydrous magnesium sulphate, phosphorous pentoxide (P_2_O_5_), sodium hydroxide (NaOH) pellets, and silica gel.

### 3.1. Synthesis of Monomers

#### 3.1.1. Synthesis of (Triphenylmethylthio)-l-cysteine [Cys(Trt)-OH] (1)

l-cysteine hydrochloride (12.12 g, 0.1 mol) was stirred in glacial acetic acid (100 mL; Acros, NJ, USA) and warmed in an oil bath at 60 °C. Triphenylmethanol (26.03 g, 0.1 mol; Sigma-Aldrich, Shanghai, China) was added, followed by boron trifluoride etherate (BF_3_·Et_2_O) (14 mL, 0.11 mol; Sigma-Aldrich, Shanghai, China). The solution was left stirring in an oil bath at 80 °C for 30 min and then cooled to room temperature. The reaction mixture was transferred to a 1 L beaker after 45 min. Ethanol (150 mL), distilled water (50 mL), and anhydrous sodium acetate (30 g) were added. The mixture was placed in a cold water bath containing ice. A white gum was separated and solidified upon dilution with distilled water (400 mL). The solid was de-agglomerated, filtered under vacuum, and washed thoroughly with excess distilled water, acetone, and diethyl ether. The obtained white solid was dried using a desiccator that contained silica gel, NaOH pellets and P_2_O_5_. The white solid was obtained with a percentage yield between 80% and 85%. The compound was spotted at *R*_f_ 0.775 via thin layer chromatography (TLC) using the following solvent system: *n*-BuOH:HOAc:EtOAc = 1:1:1, *v*/*v*/*v*. m.p. 172 °C. Analytical calculations for C_22_H_21_NO_2_S: C 72.69%; H 5.82%; N 3.85%; S 8.82%. Analysis obtained: C 73.57%; H 5.23%; N 3.11%; S 8.10%. FTIR: *v*_max_ 3242, 3056, 2925, 1634. ^1^H-NMR (400 MHz, DMSO) δ = 2.51 (t, 2H), 2.95 (d, *J* = 7.2, 1H), 2.56 (d, *J* = 6.7, 2H), 7.29 to 7.30 (m, 15H) (Figure S1).^13^C NMR (100 MHz, DMSO) δ = 175.0, 144.6, 128.1, 128.6, 127.3, 67.4, 56.9, 33.2. LC-MS: *m/z* [M^+^ + H_2_O] 381.1, [M^+^] 363.1.

#### 3.1.2. Synthesis of Cys(Trt)–OH [Cys(Trt)-OLi]lithium Salt (2)

Lithium hydroxide (0.47 g, 0.02 mol) was stirred with (triphenylmethylthio)- l-cysteine (7.2 g, 0.02 mol) in distilled water (100 mL) until a clear solution was observed. The solution was stored in a freezer at −70 °C for 24 h and then freeze dried under vacuum at −40 °C for 24 h to ensure complete drying. As the compound was hygroscopic, it was placed under extensive drying in a desiccator that contained P_2_O_5_. The white solid obtained was spotted at *R*_f_ 0.669 on TLC using the following solvent system: *n*-BuOH:HOAc:EtOAc = 1:1:1, *v*/*v*/*v*. m.p. 189 °C. Analytical calculations for C_22_H_20_NO_2_SLi: C 71.52%; H 5.45%; N 3.79%; S 8.66%. Analysis obtained: C 70.10%; H 5.10%; N 3.25%; S 8.01%. FTIR: *v*_max_ 3354, 3289, 3056, 2917, 1597. ^1^H-NMR (400 MHz, D_2_O) δ: 2.41 (d, *J* = 6.2, 2H), 2.38 (d, *J* = 7.2, 2H), 2.87 (t, 1H), 7.11 to 7.21 (m, 15H) (Figure S2). ^13^C NMR (100 MHz, DMSO) δ = 178.9, 145.1, 128.3, 128.2, 127.5, 67.2, 54.5, 33.1. LC-MS: *m/z* [(M − 1) + Na^+^] 391.2.

#### 3.1.3. Synthesis of 2,2-Difluoro-4-tritylsulfanylmethyl-1,3,2-oxazoborolidin-5-one (3)

Compound **2** (0.73 g, 0.002 mol) was stirred in 15 mL of dry tetrahydrofuran (THF) prepared by adding molecular sieve granules into THF and storing them overnight. Dry THF was bubbled with nitrogen gas for 1 h prior to the experiment. BF_3_·Et_2_O (500 μL, 0.004 mol) was added to the suspension. The mixture was warmed in an oil bath at 60 °C and then stirred for 7 h until a clear solution was formed. Progress of the reaction was monitored by thin layer chromatography (TLC). The resulting solvent was evaporated immediately under vacuum to anticipate the opening of the five-membered ring lactones. TLC analysis (ethyl acetate:petroleum ether = 4:6) produced two spots, one showing the starting material Cys(Trt)–OLi (*R*_f_ 0.669) and the other showing the oxazolidinone compound. Purification with column chromatography (ethyl acetate:petroleum ether = 4:6) produced a brown sticky oil with a percentage yield between 18% and 20%. The spot on TLC at *R*_f_ 0.455 using the same solvent system turned violet after being sprayed with ninhydrin reagent, indicating the presence of an amine group. The NMR sample was stable for at least 5 d under stringent dry conditions. FTIR: *v*_max_ 3465, 3056, 2852, 1753. ^1^H-NMR (400 MHz, CDCl_3_) δ = 2.84 to 2.85 (m, 2H), 2.87 (d, *J* = 5.2, 2H), 3.11 to 3.17 (m, 1H), 7.37 to 7.51 (m, 15H) (Figure S3). ^13^C NMR (100 MHz, CDCl_3_) δ = 170.5, 144.7, 128.2, 128.0, 127.3, 66.9, 62.1, 33.3. LC-MS: *m/z* [(M + 1) + H_2_O] 410. Data for elemental analysis could not be obtained because of the instability of the compound.

#### 3.1.4. Synthesis of (Triphenylmethyl) Thiopropionic Acid (4)

Triphenylmethanol (10.42 g, 0.04 mol), 3-mercaptopropionic acid (3.48 mL, 0.04 mol), and glacial acetic acid (100 mL) were stirred and heated to 60 °C in an oil bath. BF_3_·Et_2_O (8 mL, 0.04 mol) was added, and the reaction mixture immediately turned reddish brown. The mixture was allowed to cool to room temperature and was left stirring for another 1 h to yield a yellow precipitate. The reaction mixture was poured into distilled water (90 mL), which resulted in the deposition of a white granular solid. The deposited white solid was washed thoroughly with distilled water and diethyl ether and then recrystallised with hot ethanol to produce shiny needle-like crystals with a yield percentage of 72%. m.p. 205 °C. Analytical calculations for C_22_H_20_O_2_S: C 76.29%; H 5.78%; S 9.20%; Analysis obtained: C 75.57%; H 6.63%; S 8.79%. FTIR: *v*_max_ 3424, 3060, 2913, 1708, 776, 702 (5-adjacent aromatic CH). ^1^H-NMR (400 MHz, DMSO) δ = 2.12 (t, 2H), 2.28 (t, 2H), 7.27 to 7.28 (m, 15H aromatic) (Figure S4). ^13^C NMR (100 MHz, DMSO) δ = 170.5, 144.7, 128.2, 128.0, 127.3, 66.9, 62.1, 33.3. LC-MS: *m/z* [M^+^ + Na] 371, [(M + 1) + MeOH] 381.

#### 3.1.5. Synthesis of 3-Tritylsulfanyl-*N*-2-(3-tritylsulfanylpropionamide)-3-tritylsulfanyl Propionic Anhydride (5)

Compound **3** (0.5 g, 1.21 mmol) and **4** (0.84 g, 2.42 mmol) were stirred in anhydrous dichloromethane (80 mL) in an ice bath under an inert atmosphere before dicyclohexylcarbodiimide (0.56 g, 2.74 mmol) was introduced. Stirring was continued at 0 °C for 9 h, and the reactants were left to react overnight at temperatures between 2 and 8 °C in a refrigerator. The resulting insoluble dicyclohexylurea was filtered off, and the collected filtrate was washed sequentially with 1 mol L^−1^ NaOH, 2 M citric acid, and distilled water. The resulting solution was filtered under vacuum. TLC showed three spots, two of which corresponded to those of the starting materials. Purification with gravity column chromatography (EtOAc:petroleum ether = 1:4) yielded a white solid (*R*_f_ 0.89) with a percentage yield between 35% and 40%. m.p. 289 °C. Analytical calculations for C_66_H_57_NO_4_S_3_: C 77.35%; H 5.30%; N 1.49%; S 9.10%. Analysis obtained: C 77.38%; H 5.61%; N 1.36%; S 9.38%; FTIR: *v*_max_ 3052, 2933, 1589, 747 to 698 (5-adjacent aromatic CH). ^1^H-NMR (400 Hz, DMSO) δ = 1.15 to 1.28 (m, 8H), 1.40 (q, 1H), 2.28 (d, 1H), 2.55 (d, *J* = 5.9, 2H), 7.21 to 7.52 (m, 45H) (Figure S5). ^13^C NMR (100 MHz, DMSO) δ = 172.5, 144.4, 129.2, 128.2, 127.3, 66.2, 34.2, 26.0. LC-MS: *m/z* [M^+^ + Na] 371, [(M + 1) + MeOH] 381.

#### 3.1.6. Synthesis of 3-Mercapto-*N*-2-(3-mercaptopropionamide)-3-mercapto Propionic Anhydride (6)

A suspension of **5** (0.089 g, 0.3 mmol) in anhydrous dichloromethane (5 mL) was treated with trifluoroacetic acid (5 mL). Four drops of triethylsilane were added to the reaction mixture, which was left stirring for 5 h. The solvent was evaporated under vacuum, and the reactants were washed with copious petroleum ether, which yielded an off-white fine powder. Purification with gravity column chromatography (EtOAc:MeOH = 2:1) yielded a white compound with a percentage yield between 20 and 30%. m.p. 121 °C. Analytical calculations for C_9_H_15_NO_4_S_3_: C 35.26%; H 4.79%; N 4.37%; S 32.01%. Analysis obtained: C 36.34%; H 5.08%; N 4.71%; S 32.34%. FTIR: *v*_max_ 1712, 1528, 2929, 2635, 3309. ^1^H-NMR (400 MHz, DMSO) δ = 2.73 (d, *J* = 6.3, 1H), 2.18 (d, *J* = 6.2, 2H), 1.81 (q, 1H), 1.01 to 1.29 (m, 15H) (Figure S6). ^13^C NMR (100 MHz, DMSO) δ = 173.8, 171.0, 169.5, 53.7, 37.6, 35.7, 29.5, 23.8, 20.5. LC-MS: *m/z* [M^+^ + Na] 371, [(M + 1) + MeOH] 381.

### 3.2. Oxidation of Thiol Monomers

A mixture of trithiol cysteine derivative and 1,2-ethanedithiol was stirred in an ammonium bicarbonate buffer (0.1 mol L^−1^, pH 8.3). DMSO then was added until approximately 50% of the solids were dissolved [23]. The mixture was stirred continuously and the reaction was allowed to proceed (open to air) for 24 to 48 h. The reaction was terminated when no more thiol could be detected using sodium nitroprusside. The resultant white suspension was filtered and washed with water, dichloromethane, and methanol to produce a powdery solid. The following combinations (molar ratios) of polymers were synthesised using the trithiol cysteine derivative and 1,2-ethanedithiol (HS–CH_2_–CH_2_–SH) as starting materials.

P10 = trithiol monomer only
P11 = 1.0 trithiol monomer:1.0 dithiol monomer
P151 = 1.5 trithiol monomer:1.0 dithiol monomer
P15 = 1.0 trithiol monomer:5.0 dithiol monomer

### 3.3. Detection of Thiol Using Sodium Nitroprusside, Na_2_Fe(CN)_5_NO

A 5% *w*/*v* aqueous solution of sodium nitroprusside was prepared. A test solution sample was adjusted to pH 9 to pH 11 with 35% *w*/*v* ammonium hydrogen carbonate. A few drops of sodium nitroprusside were introduced into the test solution. A dark purple colour developed in the presence of thiols. The colour intensity gradually faded after 4 min.

### 3.4. Physical Characterisation of the Synthesised Polymers

Infrared (IR) spectra using KBr discs were generated on a Nexus FTIR (Thermo Nicolet, MN, USA) spectrophotometer.

### 3.5. Raman Spectroscopy

Raman spectra were recorded using a Jobin Yvon HR 800 UV Raman spectrometer (Lower Hutt, New Zealand). The incident laser excitation wavelength was 514.5 nm with an output of 20 mW. The samples were prepared in KBr disc, and spectra were recorded from 100 to 3000 cm^−1^ to identify *S*–*S* and *S*–*H* peaks.

### 3.6. Scanning Electron Microscope-Energy Dispersive X-ray (SEM–EDX)

The representative samples were examined using SEM. The samples were surface sputtered with gold using a sputter coater (Polaron (Fisons) SC 515 sputter coater; Fisons Instruments, Uckfield, UK) Images up to 1000× magnification were taken using a SEM microscope (LEO Stereoscan 4201, Leica Electron Optics, Cambridge Instruments Ltd., Cambridge, UK). EDX was performed using the detection microanalysis system INCA 400 (Oxford Instruments PLC, Bucks, UK). EDX measurements were made using electron beam spot sizes <50 nm.

### 3.7. Assay for Thiol

#### 3.7.1. Preparation of SØrensen’s Phosphate Buffer

To prepare 100 mL of pH 7.4 buffer, 44.2 mL of 0.06 mol L^−1^ KH_2_PO_4_ were mixed with 55.8 mL of 0.06 mol L^−1^ of Na_2_HPO_4_.2H_2_O. The resulting pH was within ±0.5 of pH 7.4 and was adjusted to the desired pH with either KH_2_PO_4_ (acidic) or Na_2_HPO_4_·2H_2_O (basic).

#### 3.7.2. Determination of Thiol Content Using Ellman’s Reagent

First, 0.1 mol L^−1^ of Ellman’s reagent was prepared in SØrensen’s phosphate buffer pH 7.4 or 8.0. A set of sample tubes, each containing 50 μL of Ellman’s reagent and 2.5 mL of SØrensen’s phosphate buffer (pH 7.4 or 8.0), then were prepared. To each sample tube, 250 μL of each standard or unknown were added. The blank (reference) was 250 μL of SØrensen’s phosphate buffer. The tubes were mixed and left standing for 15 min at room temperature to enable the thiol exchange to occur. The UV absorbance then was measured at 412 nm using 1 cm cells. Thus, the corresponding cuvettes contained the following:

**Table:** 

Sample cuvette	50 μL Ellman’s reagent
	2.5 mL SØrensen’s phosphate buffer
	250 μL sample solution
Reference cuvette	50 μL Ellman’s reagent
	2.75 mL SØrensen’s phosphate buffer

#### 3.7.3. Application of Beer-Lambert Equation

The concentration of thiol can be calculated using the Beer-Lambert equation and an ɛ_412_ value of 14,150 L mol^−1^ cm^−1^ as follows:

(1) C=A/ɛ·d

where *C* is concentration (mol L^−1^), *A* is absorbance, *d* is cell path length (1 cm), and ɛ_412 nm_ is the molar absorption coefficient in SØrensen’s phosphate buffer pH 7.4.

### 3.8. Chemical Reduction

#### 3.8.1. Reduction of Cystamine by Zinc/Acetic Acid

Cystamine dihydrochloride (0.5 g, 0.0022 mol L^−1^) and acetic acid (1.3 mL, 0.0343 mol L^−1^) were dissolved in distilled water (10 mL) and the mixture was purged with oxygen-free nitrogen for 15 min. The mixture in a three-neck round bottom flask was refluxed at 100 °C in an oil bath. Zinc dust (1.95 g, 0.03 mol L^−1^) was then added slowly while stirring [24]. Using an HPLC microsyringe, samples (10 μL) were withdrawn from the side arm and diluted to 25 mL with distilled water. The diluted sample was mixed well and filtered through a Pasteur pipette containing pre-inserted cotton wool. To measure the thiol content, 1 mL of the filtrate was tested with Ellman’s reagent (refer to Section 3.7.2).

#### 3.8.2. Reduction of Disulphide Cross-Linked Polymers by Zinc/Acetic Acid

The procedure described in Section 3.8.1 was repeated using 0.3 g of polymer from batch 1. However, the samples were withdrawn using an Eppendorf micropipette with modified disposable tips. The modification was carried out by cutting 5 mm off the tip’s drawing end using a surgical blade to widen the orifice. The sample was then diluted with SØrensen’s phosphate buffer (pH 7.4) that contained 0.006 mol L^−1^ EDTA. Next, 1 mL of the sample solution was used to measure the concentration of thiols using Ellman’s reagent. The above methods were repeated with polymers from batches 2 and 3, respectively.

### 3.9. Dissolution Studies

#### 3.9.1. Bacteria

*B. fragilis* (ATCC 25285) was obtained from the Pathology Department, General Hospital of Pulau Pinang, Penang, Malaysia.

#### 3.9.2. Preparation of Pre-Reduced, Anaerobically Sterile (Pras) Media

The guidelines of Holdeman and Moore (1973) were adopted for the preparation of PRAS media [25]. To produce a test suspension of bacterial cells free from solid particles, the *B. fragilis* Robertson’s cooked meat media (CMM) culture was subcultured. This procedure was performed by transferring 1 mL of the bacterial suspension from the supernatant of the CMM culture into 100 mL pre-reduced anaerobically sterile-Wilkens Chalgren anaerobic broth (PRAS-WCAB) medium (100 mL) using a sterile hypodermic needle and 1 mL syringe. Incubation at 37 °C for 18 h produced a stationary phase culture containing 1 mg/mL (dry weight) of bacterial cells [26].

#### 3.9.3. Preparation of Bacterial Pellets

To isolate the bacterial cells from the *B. fragilis* PRAS-WCAB medium, the test culture was centrifuged at 10,000 rpm for 2 min. The supernatant liquid was decanted to leave the bacterial pellet at the bottom of the tube. The pellet was quickly scraped off using a sterile microspatula and transferred to appropriate anaerobic medium for incubation. The anaerobic medium was continuously bubbled with nitrogen gas prior to transference of the bacterial pellet. In order to minimise the exposure of the bacterial cells to oxygen, the whole process was completed within 10 min. Each experiment consisted of pellets generated from 100 mL of WCAB *B. fragilis* test culture.

#### 3.9.4. Incubation of Polymers in Bacterial Cultures

Visking dialysis tubing (3.3 cm × 20 cm) was softened in boiling distilled water. A knot was then tied to form an open sac and the dialysis tube was rinsed in distilled water. Next, 0.4 g of a sample polymer was inserted into the visking dialysis tubing. To this, a pellet from *B. fragilis* pre-separated from 100 mL of bacterial culture and 15 mL of SØrensen’s phosphate buffer (pH 7.4) were added. A closed sac was formed by tying a knot at the open end. The sac was placed into a 100 mL conical flask (incubation vessel) containing 90 mL of SØrensen’s phosphate buffer. The mouth of the conical flask was covered and sealed with a rubber sheet and flushed with oxygen-free nitrogen via a sterile needle. The incubation was continued in a shaking water bath at 37 °C with purging of oxygen-free nitrogen continuously. Samples were collected at 5, 30, and 180 h of incubation. Two experimental controls were used: the disulphide cross-linked polymer incubated in SØrensen’s phosphate buffer without bacteria and a *B. fragilis* suspension in buffer alone without the polymer.

#### 3.9.5. Detection of the Reduction of Disulphides Using Ellman’s Reagent

The assay for thiols was carried out using Ellman’s reagent as described in Section 3.7.2.

#### 3.9.6. Statistical Analysis

The thiol concentration data for different polymers at 5, 30, and 180 h were analysed using one-way analysis of variance (ANOVA) (SPSS for Windows, Version 10.0; IBM, New York, NY, USA) [27]. *Post-hoc* analysis using Tukey’s HSD (Honestly Significant Difference) test was performed when a statistically significant difference at *p* < 0.05 was obtained. The final thiol concentrations at 180 h for the different polymers were also compared using one-way ANOVA. *Post-hoc* analysis using Dunnett’s (two-sided) test was conducted when a statistically significant difference at *p* < 0.05 was obtained.

## 4. Conclusions

Four disulphide cross-linked polymers were synthesised via DMSO-assisted oxidation of trithiol and dithiol monomers. Polymers with a tight or loose network were produced from the polymerisation of various molar combinations of trithiol and dithiol monomers, and they were reducible to varying degrees in colonic bacterial culture. Hence, the use of cross-linked disulphide-containing polymers may prove to be an effective approach to delivering drugs to the colon. The results described herein are an important contribution to the field of drug delivery, as they illustrate the possibility of using new polymers based on a disulphide backbone in colon-targeted drug delivery systems. Nevertheless, further investigations are required to conclusively show the applicability of these polymers to be used as coating materials for pharmaceutical products such as capsules or tablets for colon-targeted drug delivery.

## Supplementary Information



## Figures and Tables

**Table 1.** Percentage yield, solubility, and physical appearance of the synthesised polymers at different ratios.

Polymer	Percentage yield	Solubility	Appearance
P10	80%–85%	Insoluble	Rugged white solid
P11	87%–91%	Insoluble	White solid
P151	86%–88%	Insoluble	White solid
P15	91%–93%	Soluble in DMSO	Yellowish white powder

**Table 2.** Reduction of cystamine by zinc/acetic acid (mean, *n* = 3).

Time (min)	Thiol concentration (mol L^−1^ × 10^−5^)	% Reduction
10	2.84	49
20	5.82	101
30	5.81	101
40	5.88	102

**Table 3.** Results of statistical comparisons of thiol concentration (×10^−6^ mol L^−1^) of various molar combinations of polymers at incubation times of 5, 30, and 180 h in phosphate buffer medium containing *B. fragilis*. Mean ± SD, *n* = 6.

Polymer	5 h	30 h	180 h
P10	1.20 ± 0.126	2.01 ± 0.435	6.90 ± 0.416
P151	2.50 ± 0.146	6.03 ± 0.106	17.10 ± 0.480
P11	12.50 ± 0.757	22.40 ± 0.928	55.92 ± 1.980
P15	25.40 ± 1.120	65.00 ± 2.419	118.60 ± 1.450
Statistical analysis	*p* < 0.05	*p* < 0.05	*p* < 0.05
Tukey’s HSD test (significant)	All significantly different	All significantly different	All significantly different

**Table 4.** The thiol concentrations (×10^−6^ mol L^−1^) for incubation at 180 h in the presence of bacteria (3). Control experiments were carried out where bacteria alone, without polymer, were incubated in phosphate buffer (1) and polymer was incubated with phosphate buffer (2). Mean ± SD, *n* = 6.

Incubation medium	P10	P151	P11	P15
Bacteria only (1)	0.247 ± 0.131	0.401 ± 0.096	0.624 ± 0.082	0.848 ± 0.234
Polymer only (2)	4.800 ± 0.289	13.012 ± 1.402	23.120 ± 0.531	33.701 ± 0.696
Bacteria + polymer (3)	6.900 ± 0.416	17.100 ± 0.480	55.920 ± 1.980	118.600 ± 1.450
Statistical analysis	*p* < 0.05	*p* < 0.05	*p* < 0.05	*p* < 0.05
Dunnett’s test (2-sided) (significant)	1 & 3 2 & 3	1 & 3 2 & 3	1 & 3 2 & 3	1 & 3 2 & 3

1 & 3 and 2 & 3 were shown to be significantly different.

## References

[1] Lloyd A.W., Martin G.P., Soozandehfar S.H. (1994). Azopolymers: A means of colon specific drug delivery. Int. J. Pharm.

[2] Leopold C.S., Eikeler D. (1998). Eudragit E as coating material for the pH-controlled drug release in the topical of inflammatory bowel disease (IBD). J. Drug Target.

[3] Chourasia M.K., Jain S.K. (2004). Polysaccharides for colon targeted drug delivery. Drug Deliv.

[4] Bragger J.L., Lloyd A.W., Soozandehfar S.H., Bloomfield S.F., Marriott C., Martin G.P. (1997). Investigations into the azo reducing activity of a common colonic microorganism. Int. J. Pharm.

[5] Dollendorf C., Hetzer M., Ritter H. (2013). Polymeric redox-responsive delivery systems bearing ammonium salts cross-linked via disulfides. Beilstein J. Organ. Chem.

[6] Schact E., Wilding I.R. (1991). Process for the preparation of azo- and/or disulphide-containing polymers.

[7] Liu H., Wang H., Yang W., Cheng Y. (2013). Disulfide cross-linked low generation dendrimers with high gene transfection efficacy, low cytotoxicity and low cost. J. Am. Chem. Soc.

[8] Harada A., Matsuki R., Ichimura S., Yuba E., Kono K. (2013). Intracellular environment-resposive stabilization of polymer vesicles formed from head-tail type polycations composed of a polyamidoamine dendron and poly(l-lysine). Molecules.

[9] Wilkes G.L. (1981). An overview of the basic rheological behaviour of polymer fluids with an emphasis of polymer melts. J. Chem. Edu.

[10] Le Q.G. (1998). Novel polymers for targeting drugs to the colon. Ph.D. Thesis.

[11] Sahudin S. (2001). Novel disulphide-containing crosslinked polymer for colon-specific drug delivery. Ph.D. Thesis.

[12] Satav S.S., Karmalkar R.N., Kulkarni M.G., Mulpuri N., Sastry G.N. (2006). Formation of linear polymers with pendant vinyl groups via inclusion complex mediated polymerization of divinyl monomers. J. Am. Chem. Soc.

[13] Tam J.P., Wu C.R., Liu W., Zhang J.W. (1991). Disulfide bond formation in peptides by dimethyl sulfoxide. Scope and applications. J. Am. Chem. Soc.

[14] Bulaj G. (2005). Formation of disulfide bonds in proteins and peptides. Biotechnol. Adv.

[15] Sim J.H., Yamada K., Lee S.H., Yokokura S., Sato H. (2007). Synthesis and characterization of triphenylamine derivatives by oxidative polymerization. Synth. Met.

[16] Sim J.H., Ueno E., Natori I., Ha J., Sato H. (2008). Oxidation polymerization of *N*-butyl-*N,**N* diphenylamine (BDPA) and *N*-4-butylphenyl-*N,**N*-diphenylamine (BTPA). Synth. Met.

[17] Wallace T.J., Mahon J.J. (1964). Reactions of thiols with sulfoxides. II. Kinetics and mechanistic implications. J. Am. Chem. Soc.

[18] Gray W.R., Luque F.A., Galyean R., Atherthon E., Sheppard R.C., Stone B.L., Reyes A., Alford J., McIntosh M., Olivera B.M. (1981). Conotoxin GI: Disulfide bridges, synthesis and preparation of iodinated derivatives. Biochemistry.

[19] Snow J.T., Finley J.W., Friedman M. (1975). Oxidation of sulfhydryl groups to disulfides by sulfoxides. Biochem. Biophys. Res. Commun.

[20] Diaz F.R., Sanchez C.O., del Valle M.A., Radic D., Bernede J.C., Tregouet Y., Molinie P. (2000). Synthesis, characterization and electrical properties of poly[bis-(2-aminophenyl)disulfide] and poly[bis-(2-aminophenyl)diselenide] Part II. XPS, ESR, SEM and conductivity study. Synth. Met.

[21] Scheiba F., Benker N., Kunz U., Rzoth C., Fuess H. (2008). Electron microscopy techniques for the analysis of the polymer electrolyte distribution in proton exchange membrane fuel cells. J. Power Sources.

[22] Kimura Y., Makita Y., Kumagai T., Yamane H., Kitao T. (1992). Degradation of azo-containing polyurethane by the action of intestinal flora: Its mechanism and application as drug delivery system. Polymers.

[23] Otaka A., Koide T., Shide A., Fujii N. (1991). Application of dimethylsulphoxide (DMSO)/trifluoroacetic acid (TFA) oxidation on the synthesis of cystine-coating peptide. Tet. Lett.

[24] March J. (1992). Advanced organic chemistry: Reactions, mechanisms and structures.

[25] Holdeman L.V., Moore W.E.C. (1973). Anaerobe laboratory manual.

[26] Andreas D., Steffen D., Markus B., Stefan S., Matthias O., Sebastian S., Patrick S., Alexandra M., Masayuki W., Tadashi T. (2013). Protein tyrosine nitration and thiol oxidation by peroxynitrite—Strategies to prevent these oxidative modifications. Int. J. Mol. Sci.

[27] SPSS http://www-01.ibm.com/software/analytics/spss/.

