# Supplementary material for: Synthesis, Characterisation, and Evaluation of a Cross-Linked Disulphide Amide-Anhydride-Containing Polymer Based on Cysteine for Colonic Drug Delivery
Source: Int J Mol Sci. 2013 Dec 18;14(12):24670–91. doi: 10.3390/ijms141224670 (PMC3876135; doi:10.3390/ijms141224670)
Supplement: Supplementary file 1 [file ijms-14-24670-s001.pdf]

Supplementary Information

Figure S1. ^{1}H-NMR spectrum of compound **1**.

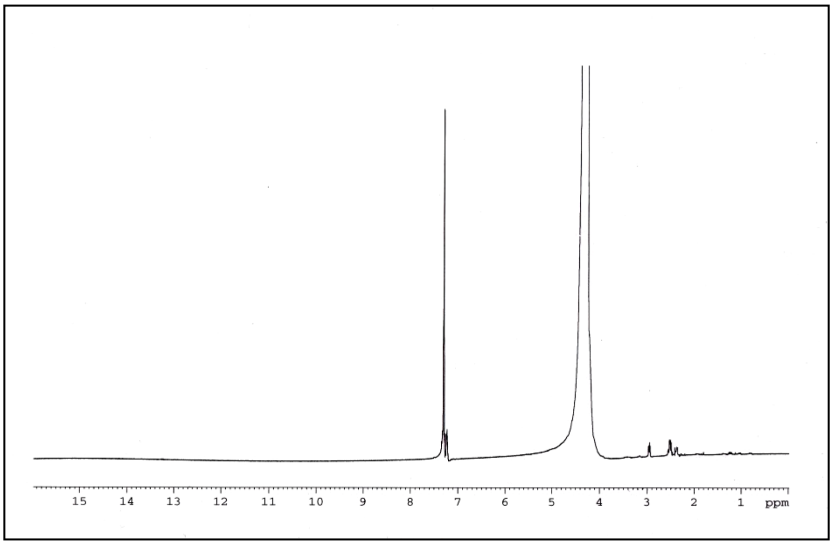

Figure S2. ^{1}H-NMR spectrum of compound **2**.

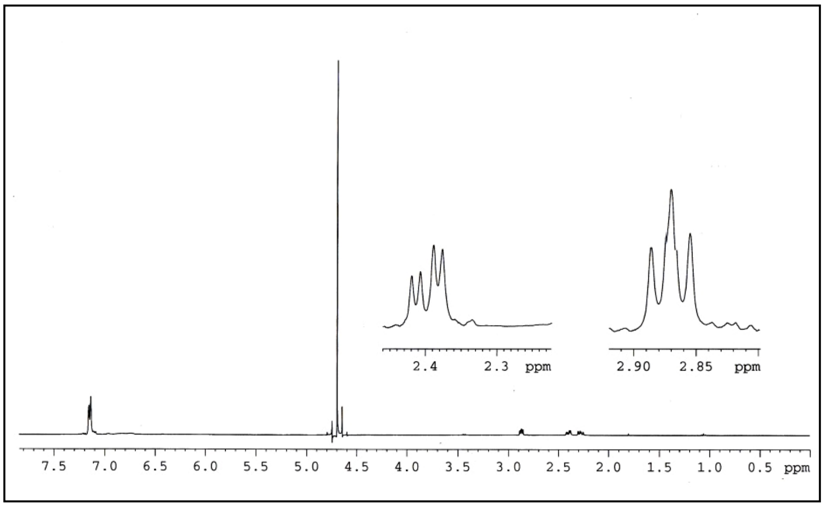

Figure S3. ^{1}H-NMR spectrum of compound **3**.

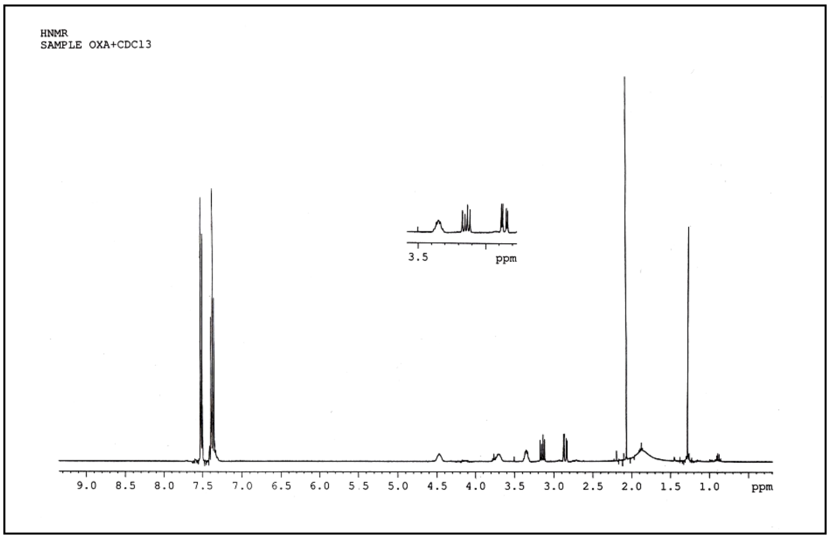

Figure S4. ^{1}H-NMR spectrum of compound **4**.

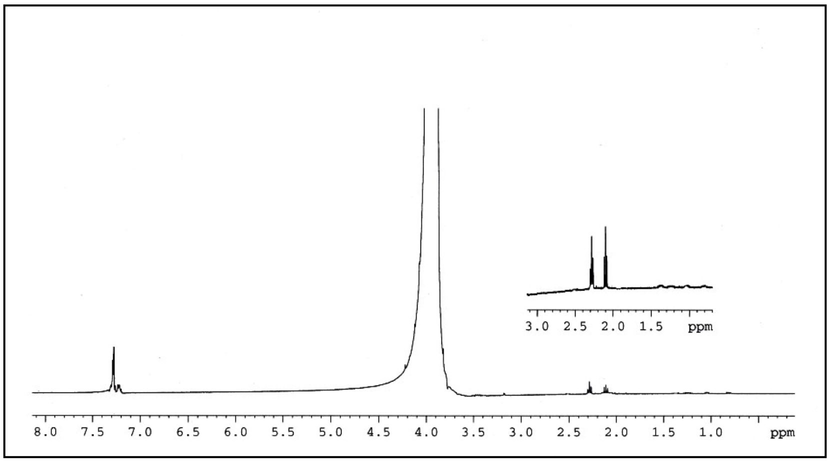

Figure S5. ^{1}H-NMR spectrum of compound **5**.

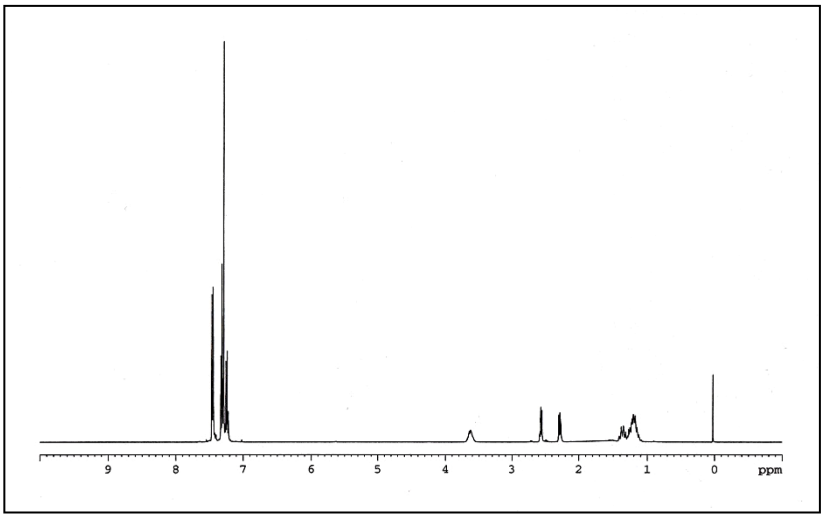

Figure S6. ^{1}H-NMR spectrum of compound **6**.

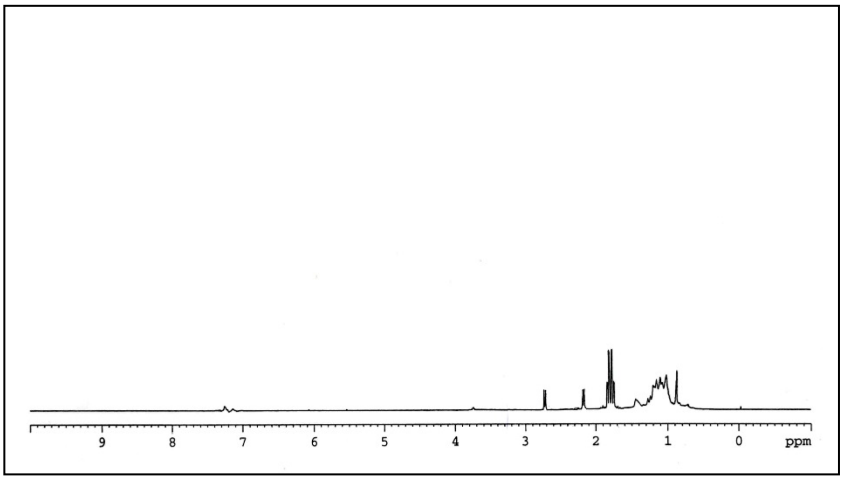

© 2013 by the authors; licensee MDPI, Basel, Switzerland. This article is an open access article distributed under the terms and conditions of the Creative Commons Attribution license (http://creativecommons.org/licenses/by/3.0/).
